# Prone-lateral access to the lumbar spine: single-level corpectomy with approach discussion

**DOI:** 10.3171/2022.3.FOCVID2216

**Published:** 2022-07-01

**Authors:** Lauren E. Stone, Luis Daniel Diaz-Aguilar, David Rafael Santiago-Dieppa, William R. Taylor, Andrew D. Nguyen

**Affiliations:** Department of Neurological Surgery, University of California, San Diego, California

**Keywords:** deformity, navigation, fluoroscopy, prone, lumbar spine

## Abstract

The lateral lumbar interbody fusion has evolved as newly envisioned access corridors become feasible with technological advances. Prone lateral access has evolved as a single-access approach to combine the benefits of minimally invasive surgery with direct and indirect decompression of the neural elements with synergistic anterior and posterior column correction. In this video, the authors discuss the pearls, pitfalls, and adjuvant technologies they use in a high-volume prone lateral center via case demonstration of a prone lateral corpectomy.

The video can be found here: https://stream.cadmore.media/r10.3171/2022.3.FOCVID2216

## Transcript

The lateral lumbar interbody fusion has evolved as newly envisioned access corridors become feasible with technological advances. Prone lateral access has evolved as a single-access approach to combine the benefits of minimally invasive surgery with direct and indirect decompression of the neural elements with synergistic anterior and posterior column correction.^1^ As our experience with this approach has evolved, so has our understanding of the challenges inherent in single-position surgery. In this video, we discuss the pearls, pitfalls, and adjuvant technologies we use in a high-volume prone lateral center.

### 1:00 Anatomical Considerations for Candidacy.

Choosing anatomically ideal candidates for prone lateral access requires evaluation of plain films and quality MRIs. As with all surgeries, safe access is predicated on knowledge of the structures in and around the operative corridor.^2–5^

In this, prone lateral surgeons are required to adopt a general surgeon mentality when evaluating the retroperitoneal space, accessing the local vasculature and neural elements. Bony anatomy including vertebral body shape, transitional levels, and iliac crest morphology are necessary to generate a 3D understanding of the access corridor.

### 1:36 Overview Clinical Pearls by Key Step.

Throughout this video, several themes will emerge that will significantly improve prone lateral access workflow. (1) Efficient use of fluoroscopy, which significantly improves workflow as the access corridor is most familiar in a true parallel-to-floor position. (2) Maintaining a healthy respect for gravity. Gravity’s pull can both help and harm access, benefitting retroperitoneal dissection while pulling the docking system suboptimally downward. Careful attention to shim placement in this position is therefore important.

### 2:11 Complications.

Our use of saphenous SSEP mitigates damage to the lumbar plexus from traction in the lateral position.^4^ Damage to anterior structures is mitigated with optimal positioning, time spent understanding imaging, and awareness of gravitational forces.

### 2:27 Clinical Case Presentation.

We will discuss pearls and pitfalls for the prone lateral corpectomy through a case example. The patient is a 68-year-old female who sustained a ground-level fall 4 months prior, now presenting with severe low-back pain and shooting pains down her right buttock and leg. Physical exam revealed no gross abnormalities in strength or sensation, but marked tenderness to the mid-lower back and a general inability to ambulate due to pain.

### 2:53 Preoperative Imaging.

MRI and x-ray from an outside hospital revealed a burst fracture at lumbar 3 causing right centric compression of the thecal sac and exiting nerve roots without gross disturbance of lumbar lordosis.

### 3:04 Proposed Operative Plan.

Given her overall clinical picture and imaging findings, she was determined to be a candidate for a prone lateral corpectomy with posterior decompression and fusion across the corpectomy level. For the purposes of this video, we will focus on the first two components of this procedure.

### Procedure Stage 1: Lateral Access and Corpectomy

#### 3:23 Positioning.

The patient is positioned prone, as demonstrated on this cadaveric model. Note the custom lateral bolsters and tape simultaneously resist lateral forces while allowing manual distraction of the rib-hip angle. Also note the strict parallel position to the floor. This is critical for the access surgeon to internalize, especially if slight oblique angulation is necessary for ergonomic ease.

#### 3:46 Incision.

The ribs and iliac crest are marked. The planned incision is placed slightly posterior to a typical LLIF incision in order to account for gravity. This can either be found with preplanned robotic navigation or fluoroscopy. Here we demonstrate incision marking via the latter. A line is made through the midpoint of the disc space followed by the posterior, then midpoint, of the vertebral body. The incision is then marked as a diagonal across a natural Langer line.

#### 4:14 Retroperitoneal Access.

Retroperitoneal access is made with sweeping motions, feeling for the iliac crest inferiorly as a guide point. A posterior-to-anterior trajectory is safest, pushing the abdominal contents with gravity downward rather than lateral. Palpation of the transverse process confirms medial extent.

#### 4:32 Dilation.

And the initial dilator is then passed over the docked hand. Position at the disc space is confirmed on AP and lateral fluoroscopy. This is then held with a K-wire.

#### 4:41 EMG/Access System.

Directional EMG is then used to guide dilation. Once satisfied, the access system is then placed over the final dilator in a similar fashion and then docked to the patient. Spot fluoroscopy is performed to confirm the site. Directional EMG is then performed to confirm the position.

#### 4:59 Corridor View, Retractor Optimization, SSEP.

The first view into the working corridor is now seen, ideally, and endplate-to-endplate view with the posterior disc space in site. The dilator is carefully opened to maximize this corridor. We also use saphenous SSEP as a timely gauge for retractor removal. We have found that this better approximates neural injury over standard posterior tibial SSEPs given that the femoral nerve is at a higher risk of apraxia during this approach.^4^

#### 5:25 Discectomy/Corpectomy.

We now localize for our corpectomy confirming our disc spaces superiorly and inferiorly. The discectomy is performed efficiently with care to maintain an orthogonal trajectory and to not push through the contralateral annulus. The vertebral body is also carefully removed under fluoroscopic guidance. The trial is sized and the implant is placed and expanded.

### Procedure Stage 2: Posterior Percutaneous Pedicle Screw Instrumentation

#### 5:49 Perc Screws.

Perc screws are performed in standard fashion with the lateral incision kept open in case extra extension of the cage is necessary after posterior distraction. In this case, no adjustments were made. All incisions were then closed in standard fashion.

#### 6:06 Clinical Conclusion.

The patient was discharged on postoperative day 6, ambulating with a front wheel walker without new neurological deficit. Postoperative films are shown here.

#### 6:13 Evolving Indications.

Careful preoperative planning, knowledge of technology available, and capacity to mitigate inherent procedural pitfalls will continue to allow surgeons to maximize the prone lateral corridor for lumbosacral pathology.

## Disclosures

Dr. Taylor is a consultant for AlphaTech. Dr. Nguyen is a consultant for Globus.

## Author Contributions

Primary surgeon: Taylor, Nguyen. Assistant surgeon: Stone, Santiago-Dieppa. Editing and drafting the video and abstract: all authors. Critically revising the work: Stone, Diaz-Aguilar, Santiago-Dieppa, Nguyen. Reviewed submitted version of the work: all authors. Approved the final version of the work on behalf of all authors: Stone. Supervision: Santiago-Dieppa, Taylor.
